# Exemption of the Cherokee Indians and Africans from Insanity

**Published:** 1845-05

**Authors:** 


*Exemption of the Cherokee Indians and Africans from Insanity.*—Dr. Lillybridge, of Virginia, who was employed by the Government as the medical officer to superintend the removal of the Cherokee Indians, in 1827-28 and 9, and saw more than twenty thousand Indians, and inquired much about their diseases, informs us he never saw or heard of a case of insanity among them.

Dr. Butler, who has been a devoted missionary and physician among the Cherokees for about a quarter of a century, informs us, in a recent letter, that he has yet seen no case of decided insanity among them, though he has occasionally seen them delirious when sick of other diseases; and adds, that an intelligent chief, a man now 80 years old, told him that "he had never known a case of insanity among his people, such as he had seen in the Hospital in Philadelphia."

Insanity is rare, we believe, among the Africans. Cinquez, and other of the *Amistad* Negroes, when in this country a few years since, visited the Retreat for the Insane at Hartford, Ct,, and saw many of the patients there. They informed the writer of this article, that insanity was very rare in their native country. Most of them had never seen an instance. Cinquez stated however that he had seen one case.—*American Journal of Insanity,*

